# Cryopreservation of testis tissues and in vitro spermatogenesis

**DOI:** 10.1007/s12522-015-0218-4

**Published:** 2015-08-05

**Authors:** Tetsuhiro Yokonishi, Takehiko Ogawa

**Affiliations:** ^1^ Department of Urology Yokohama City University Graduate School of Medicine 236‐0004 Yokohama Japan; ^2^ Laboratory of Proteomics, Institute of Molecular Medicine and Life Science Yokohama City University Association of Medical Science 236‐0004 Yokohama Japan

**Keywords:** Cryopreservation, In vitro spermatogenesis, Male infertility, Pediatric cancers, Testis tissues

## Abstract

Cancer treatments, either chemo‐ or radiotherapy, may cause severe damage to gonads which could lead to the infertility of patients. In post‐pubertal male patients, semen cryopreservation is recommended to preserve the potential to have their own biological children in the future; however, it is not applicable to prepubertals. The preservation of testis tissue which contains spermatogonial stem cells (SSCs) but not sperm would be an alternative measure. The tissues or SSCs have to be transplanted back into patients to obtain sperm; however, this procedure remains experimental, invasive, and is accompanied with the potential risk of re‐implantation of cancer cells. Recently, we developed an organ culture system which supports the spermatogenesis of mice up to sperm formation from SSCs. It was also shown that the tissues could be frozen for later sperm production, which resulted in the generation of offspring. Thus, it could be useful as a clinical application for preserving the reproductive potential of male pediatric cancer patients. The establishment of an optimized cryopreservation method and the development of a culture system for human testis tissue are expected in the future.

## Introduction

Owing to recent progress in medical treatments for cancer patients, long‐term survival and even cure have become possible for young cancer patients. Therefore, an increasing number of cancer survivors exist who are suffering from the adverse effects of treatments they underwent. Infertility is one of the side‐effects and could be the most influential factor affecting the psychological aspects of their lives [1]. Thus, not only cancer treatment but also fertility preservation has become important for cancer patients, especially young people [2]. Our aim is to review the up‐to‐date literature regarding the impact of cancer treatment on male fertility and its prevention, including semen cryopreservation. We also discuss experimental challenges and our method of testis tissue cryopreservation and in vitro spermatogenesis for preserving the fertility of pre‐adolescent male cancer patients.

## Interest in fertility of male cancer survivors

A survey involving young cancer patients aged 14–40 years at the time of diagnosis showed that 51 % of them wanted to have their own children in the future. Moreover, this desire was naturally stronger in patients who did not have children at the time of inquiry, with 77 % of them answering in this way [3]. Another study demonstrated that an even higher proportion of male patients (70 %) wanted to have children in the future after finishing the treatment [2]. Today, clinicians need to know the disposition of such patients, discuss the risk of infertility due to the treatments, and present options for fertility preservation prior to the treatments [4]. In 2006, the American Society of Clinical Oncology (ASCO) recommended that clinicians include fertility preservation measures in the cancer treatment scheme for each patient [5]. Such recognition was not fully adopted among clinicians at that time. A study showed that among 111 testis cancer survivors aged 18−45 years at the time of diagnosis (from 2003 to 2007), 36 men (32 %) had not been informed about semen cryopreservation before treatment [6]. A study in 2010 using a questionnaire survey involving 6,224 pediatric cancer survivors revealed that they had significantly lower chances of having children—56 % compared to their siblings [7]. This does not directly reflect their reproductive ability and may be associated with their lifestyle as a cancer survivor as a whole, i.e., it emphasizes the impact of the treatment that they underwent on their later lives. Fertility after cancer treatment is now becoming a major concern among young cancer patients and their families.

## Gonadal toxicity of cancer therapy

In the mature testis, spermatogonia divide actively to produce huge numbers of cells as the source of sperm. Chemo‐ and radiotherapies preferentially impair dividing cells, including spermatogonia and spermatocytes in the testis. Human spermatogonia are classified into type A (undifferentiated) and type B (differentiated). Type A spermatogonia are further sub‐classified into A_dark_ and A_pale_ based on their nuclear features on hematoxylin staining. A_pale_ spermatogonia divide actively, while A_dark_ are relatively dormant and thought to be stem cells based on studies in primates [8, 9, 10]. On the other hand, type B spermatogonia divide more actively to differentiate into spermatocytes. Thus, type B spermatogonia are more sensitive to cytotoxic treatments than type A. It has been reported, however, that even low doses of chemo‐ or radiotherapy can induce apoptosis of not only type B but also type A spermatogonia [11, 12, 13, 14]. Naturally, treatment with higher doses will destroy spermatogonia, leading to a Sertoli‐only state of the testis and inducing longer periods of infertility in patients [15].

The effects of cytotoxic therapies can appear not only as a reduction of sperm number, i.e., oligozoospermia or azoospermia, but also as chromosomal aberrations of sperm. The chromosome anomalies include aneuploidy, partial defects, and translocation, which are triggered mostly during the meiotic phase of spermatocytes. Thus, their incidence rises rapidly after treatment and then reportedly declines over time. The appearance of aneuploidy, for example, was transient and reported to take approximately 100 days to return to the pre‐treatment level [16]. Other reports, however, stated that aneuploidy induced by cancer therapy lasted for 1–2 years or even longer [17, 18], which indicates that spermatogonia along with spermatocytes were influenced by cytotoxic treatments.

Because alkylating agents, such as cyclophosphamide, affect both dividing and dormant cells [19], they induce marked spermatogenic impairment. A study which examined 355 male patients with Hodgkin's lymphoma 12 months after treatment reported that follicle‐stimulating hormone (FSH), an increased level of which indicates impaired spermatogenesis, was in the higher range in only 8 % of patients who were treated with regimens involving non‐alkylating agents alone, such as ABVD (doxorubicin, bleomycin, vinblastine, and dacarbazine) or EBVP (epirubicin, bleomycin, vinblastine, and prednisone). On the other hand, high FSH was observed in 60 % of patients treated with a regimen including alkylating agents, such as MOPP (mechlorethamine, vincristine, procarbazine, and prednisone), or MOPP/ABV (doxorubicin, bleomycin, and vinblastine), or BEACOPP (bleomycin, etoposide, doxorubicin, cyclophosphamide, vincristine, procarbazine, and prednisone) [20]. It was also reported that FSH recovered to a normal range in 82 % of patients, taking a median of 19 months in cases treated without alkylating agents. Meanwhile, it recovered in only 30 % of patients and took a median of 27 months in cases treated with alkylating agents [20]. Therefore, the adverse effect of alkylating agents on spermatogenesis is severe and recovery takes a long time or may not be achievable. The most effective measure to mitigate their damage is naturally to avoid their use by replacing them with other agents if possible. It is also important to know the threshold doses of agents, if available, whereby a higher does is likely to induce irreversible damage on spermatogenesis. For example, it has been reported that cyclophosphamide with a total dose of >7.5 g/m^2^ induced permanent infertility in approximately 90 % of patients, while doses <7.5 g/m^2^ allowed recovery to a normospermic level in approximately 70 % of patients [21].

Some agents other than alkylating ones are also toxic to gonads. Among others, cisplatin, a platinum agent, is frequently used for many cancers, including testicular cancer. Analysis of 178 testicular cancer patients who underwent chemotherapy using cisplatin identified 52 % patients showing azoospermia 2 years after therapy, and 20 % remained so at 5 years [22]. Again, the total cumulative dose of cisplatin administered could be an indicator of whether azoospermia is transient or permanent. When the cumulative dose of cisplatin was <400 mg, being roughly equivalent to 4 courses of state‐of‐the‐art treatment, the damage is likely to be reversible [23].

Radiation, similar to alkylating agents, markedly impairs spermatogenesis. Total body irradiation, performed as a conditioning measure for bone marrow transplantation (BMT), significantly damages germ cells. After treatment with total body irradiation at a total dose of either 10 or 13 Gy, azoospermia was noted in 41 of 48 patients (85 %), leaving the remaining 7 with oligozoospermia [24]. These data signify the impact of irradiation on germ cells, considering that pre‐treatment for BMT with cyclophosphamide alone rendered only 1 out of 10 patients with azoospermia in the same study [24]. Regional irradiation as well as total body irradiation could induce testicular damage as some doses of radiation can be scattered outside the targeted region. In the case of abdominopelvic irradiation, approximately 1–2 % of doses aimed at the tumor were estimated to reach the testes [25]. Damage of germ cells directly induced by irradiation is proportional to the dose. It was reported that doses as low as 0.1 Gy reaching the testis resulted in the cessation of spermatogenesis [26]. A dose of 2–3 Gy causes long‐term azoospermia, and >6 Gy can deplete the spermatogonial stem cell pool and lead to permanent infertility [26, 27].

In the testis, Leydig cells are also affected by radiation when the dose is high enough [28]. A clinical study reported the gonadal function of 15 boys with acute lymphoblastic leukemia who had received testicular irradiation. Doses to the testes were 12 Gy in 12 cases, 15 Gy in 1 case, and 24 Gy in 2 cases. All patients who received 12 or 15 Gy showed normal Leydig cell function, but the 2 patients who received 24 Gy suffered from depletion of testosterone and required androgen replacement treatment [29].

Alleviation of radiation‐induced damage to the testis could be achieved only by minimizing the dose as low as possible. This can be performed by shielding the testes or reducing the radiation dose in the first place while maintaining a sufficient effect. In the past, however, gonadal protection through hormonal suppression was attempted, based on the assumption that germ cells become less sensitive to cytotoxic treatments when they are rendered to be mitotically quiescent. In fact, this strategy was reported to be effective in animal experiments using rats [30]; however, trials in clinical settings did not show the same results [31, 32, 33]. A study reported that gonadotropin‐releasing hormone agonist treatment of seminoma patients who underwent irradiation of their testes did not show protective effects when judged on serum levels of FSH, luteinizing hormone, and testosterone [31].

## Semen cryopreservation

As a means to preserve fertility, semen cryopreservation prior to gonadal toxic therapy has been established for post‐pubertal patients. When micro‐insemination was not available, cryopreserved sperm were used for artificial insemination. This was not successful in many cases because the freezing and thawing procedure made the sperm less motile and reduced their number. In addition, cryopreserved semen would have been sufficient for only a single or a few insemination procedures. Now that the intracytoplasmic sperm injection (ICSI) procedure has been established and become popular, cryopreserved sperm can be applied with this technique. It was reported that the condition of sperm, whether freshly obtained or cryopreserved, does not cause a difference in the outcome, e.g., the pregnancy rate [34, 35, 36]. Thus, semen cryopreservation was validated as a method to preserve the possibility of having one's own biological child in the future. Today, clinicians need be aware of it and are requested to check if their patients are eligible for the procedure. Particularly in the case of patients in puberty, clinicians need to address if their patients have begun spermatogenesis, and whether or not they can ejaculate. A study reported that sperm in urine were noted among 20 % of 11‐ to 12.5‐year‐old boys in Germany [37]. Other studies reported that boys started masturbation at an average age of 12 years and 80 % of boys did so by the age of 13 years, suggesting that boys aged ≥12 years in most cases can collect semen by masturbation [38, 39]. Other studies reported that all pubertal boys with testis volumes >10–12 mL are encouraged to collect semen samples for preservation before cancer therapy [39, 40].

Although semen collection is recommended prior to the initiation of treatment, a study reported that 20 % of patients had frozen their sperm during the course of cancer treatment [41]. The fidelity of these sperm is at risk because the cancer treatment would damage the DNA of germ cells which could be carried up to sperm formation. Thus, semen cryopreservation is definitely recommended to be performed before treatment starts [41, 42].

Cryopreserved sperm has been used and resulted in the birth of babies. One report stated that 29 patients who preserved their semen underwent 87 cycles of reproductive procedures in total, including 42 cycles of intrauterine insemination (IUI), 26 of in vitro fertilization (IVF), and 19 of ICSI, resulting in a pregnancy rate of 18.3 % (7 % IUI, 23 % IVF, and 37 % ICSI) of which 75 % led to live births (100 % IUI, 83 % IVF, and 57 % ICSI) [43]. Regarding the preservation period, a case report proved the fertility of sperm cryopreserved for 28 years by achieving a live birth using micro‐insemination [44]. Thus, sperm can be quite resilient and cryopreservation secures them for several decades.

As mentioned above, semen cryopreservation is a useful and established method for post‐pubertal patients to preserve their reproductive potential. However, it is not applicable for pre‐pubertals. Therefore, cryopreservation of SSCs or testis tissue en bloc would be an option. There are various ways to obtain sperm from those samples, e.g., spermatogonial transplantation, testis tissue grafting, and in vitro spermatogenesis.

## Spermatogonial stem cell transplantation

Spermatogonial transplantation was developed by Ralph L. Brinster in 1994 [45]. It was also found soon after that cryopreservation of SSCs was possible and sperm production from a frozen sample was performed by transplantation into the seminiferous tubules of host mice [46]. The procedure of spermatogonial transplantation was extensively studied in mice and extended to rats as host animals [47]. Its application was limited to rodents and interspecific transplantation was also limited between species phylogenetically close to each other, such as rats and mice or hamsters and mice [48, 49]. Recently, however, it was reported that the technique of spermatogonial transplantation was modified and applied to rhesus macaques. In this case, the donor and host combination was autologous or allogenic. The authors reported that the injected SSCs differentiated up to sperm in the seminiferous tubules in both autologous and allogeneic combinations [50].

Spermatogonial transplantation to the human testis was investigated in the past. With the injection of a dye solution into the seminiferous tubules of human testis, it was reported that 55 % of the total tubular lumen was stained [51]. Another study reported that ultrasound‐guided injection was useful in the human testis [52]. Auto‐transplantation of the germ cells of 7 adult patients with Hodgkin's lymphoma was attempted in a study. The testicular cells of patients were harvested and cryopreserved before treatment, and were transplanted back into the seminiferous tubules of each patient after treatment. The outcome of this study, however, has not been reported [53, 54]. Although these reports suggest that the injection of cells into human seminiferous tubules is technically possible, the production of sperm and harvesting them might not be sufficiently feasible to be clinically applicable. One of the options which could make spermatogonial transplantation more generally applicable is to use purified SSCs. This could be achieved if SSCs were cultured for propagation. Indeed, it is possible in the case of mouse SSCs; however, the in vitro propagation of human SSCs is not yet feasible.

## Testis tissue grafting

Testicular grafting was found to be effective in inducing spermatogenesis in a small piece of immature testis tissue [55]. The main strength of this method is that it could be applicable to diverse mammalian species, using nude mice as a host animal [55, 56]. Pre‐pubertal mouse, rabbit, sheep, and pig testicular tissue surprisingly survived after grafting in the dorsal subcutis of the host and produced sperm, some of which had their fertility proven by micro‐insemination [57, 58]. A study reported that the grafting of testis tissue from immature 13‐month‐old rhesus monkeys into host mice resulted in the acceleration of testicular maturation and production of fertility‐competent sperm in testis xenografts. Rhesus monkeys start spermatogenesis at 3–4 years old, but grafted testis tissues produced sperm in 7 months [59]. However, in the case of autologous grafting of rhesus monkey testis tissues, a study reported different results. One hundred and thirty pieces of cryopreserved testis tissue derived from 5 rhesus monkeys, aged 30–49 months, in which spermatogenesis proceeded up to preleptotene stermatocytes, were autologously grafted in the subcutis of the scrotum, shoulder, back, and arms. At 5 months after implantation, sperm, round spermatids, and pachytene spermatocytes were detected in 2 (1.5 %), 1 (0.8 %), and 4 (3.1 %) fragments, respectively. In addition, the graft recovery rate was only 0–7 % [60]. Therefore, this study concluded that in order to compensate for this low recovery rate, the amount of testis tissue grafted should be as much as possible, such as one whole testis.

To our knowledge, there has not been an autograft experiment using human testis. There are, however, several studies using immunodeficient mice as host animals [61, 62]. Testis tissue fragments measuring 0.5–1 mm^3^ from 32–40‐year‐old patients with obstructive azoospermia, non‐obstructive azoospermia, and testicular cancer were grafted into nude or SCID mice. It was observed that most seminiferous tubules underwent hyalinization changes within a week and germ cells disappeared by 14 weeks [61]. In another study using 4 mm^3^ testis tissue obtained from patients who underwent reversal surgery for vasectomy, 74 tissue pieces were grafted to the backs of SCID‐NOD mice. All grafts showed severe sclerotic change and germ cells also disappeared in most cases, progressing only up to the Sertoli cell‐only state. Only 16 tissues (21.6 %) maintained some spermatogonia [62]. On the other hand, a study reported that xenografting of testicular tissue from an infant human donor resulted in accelerated testicular maturation [63]. Taken together, testis tissue grafting could be a means to obtain sperm from immature testis tissues, but its efficiency is not stable and may not be satisfactory. In the case of adult tissue, the grafting cannot maintain spermatogenesis, being rather prone to degeneration.

## Cryopreservation technique of testis tissue

We report a possible new strategy to preserve fertility in pre‐pubertal male cancer patients. As we developed an organ culture method which can support the complete spermatogenesis of neonatal or pup mice, we applied this technique and examined several methods of cryopreservation. Here, we review cryopreservation and present our results.

Successful cryopreservation depends on the choice of cryoprotectant and its concentration, and procedures both for freezing and thawing. In a study using a slow‐freezing procedure for pre‐pubertal human testis tissues, dimethyl sulfoxide (DMSO) was reported to be the most favorable among the cryoprotectants tested, including ethylene glycol, propanediol, and glyceol, based on histological observation regarding the maintenance of its architecture along with the viability of constituent cells including spermatogonia, Sertoli cells, and those in the interstitium [64, 65]. In addition, the cryopreservation of immature rhesus monkey testis tissues with DMSO showed the resumption of spermatogenesis at xenografting [66]. These reports along with others demonstrated the superiority of DMSO as a cryoprotectant for testis tissue. It was recently reported that DMSO added to sucrose appears to be a choice of cryoprotectant for human testicular tissues, with either a controlled or uncontrolled slow‐freezing protocol [67]. Some previous studies have used DMSO plus sucrose for testis tissue cryopreservation, although its advantageous effects were not clearly demonstrated [68, 69, 70].

There are some inconveniences regarding the slow‐freezing method in clinical settings because it generally needs a programmable freezer and is time‐consuming. In this regard, the vitrification method is attractive. Vitrification uses solution with a higher osmolality than those used for slow freezing, and samples were placed immediately into liquid nitrogen, which makes the whole procedure able to be completed in a short period. Vitrification has been used for oocyte cryopreservation in clinics for nearly a decade. Recently, its application to testis tissue or spermatogonia was reported to be successful [67, 71, 72]. These studies showed the favorable maintenance of human testis tissue, on the basis of histological observation, by the vitrification method. At this moment, it remains unclear which method (slow freezing or vitrification) is superior for testicular tissue cryopreservation.

## In vitro spermatogenesis of cryopreserved mouse testis tissues

As stated above, spermatogonial transplantation and testis tissue grafting could facilitate the production of sperm from cryopreserved testis tissues. Both methods, however, are accompanied by marked problems when considering clinical application. Two serious problems in particular are the invasiveness of the procedure, whether cell transplantation or tissue grafting, to patients and, more seriously, the cryopreserved samples might contain malignant cells of the patient's original disease. The transplantation or grafting of such samples back into the body of each patient could lead to the re‐introduction of malignant cells. This risk seems to be very small and negligible in most cases. Animal experiments, however, demonstrated that as few as 20 leukemia cells were sufficient to cause a leukemic state in rats after introduction into seminiferous tubules of the host [73]. Cell‐sorting methods, such as FACS, could be a measure to prevent such re‐inoculation of malignant cells, but it is not reliable in reality [74, 75]. Xeno‐transplantation or xeno‐grafting would circumvent the re‐introduction of malignant cells, but they have their own problems and concerns. One such concern is that a patient's sperm produced in an animal's body might carry substances of animal origin, viruses, or DNA fragments unique to the animal [76].

Recently, our laboratory developed an organ culture system for spermatogenesis and we succeeded in obtaining functional sperm in neonatal mouse testis tissues in vitro [77]. We then applied this culture method to cryopreserved testis tissues. Spermatids and sperm were obtained in samples cryopreserved by either slow‐freezing or vitrification methods; those haploids were used for microinsemination using round spermatid injection and ICSI, respectively, leading to offspring. They grew healthily and produced the next generation by natural mating [78]. This strategy, cryopreserving testis tissue followed by in vitro spermatogenesis, overcomes the problems of the other methods, i.e., the invasiveness of the procedure to patients, and the risk of re‐implantating cancer cells.

## Conclusion

The infertility of male cancer survivors who have undergone cytoablative therapies is a crucial problem. Physicians are expected to provide patients with information about infertility as one of the late‐occurring adverse effects of treatment and on preventive measures. For pre‐adolescent male patients, we expect testis cryopreservation and our culture system to become a competent fertility‐preserving method in the future; however, our culture method is still only applicable to mouse testis tissues. Thus, optimization of culture conditions for human tissue is necessary. Further refinements of the cryopreservation technique are also required. Through these improvements, this strategy may become a method to protect and preserve the reproductive ability of young males, and may be applicable to pediatric cancer patients in the future.

## Compliance with the ethical standard

### Conflict of interest

The authors have no conflict of interest.
